# Structural Aspects and Prediction of Calmodulin-Binding Proteins

**DOI:** 10.3390/ijms22010308

**Published:** 2020-12-30

**Authors:** Corey Andrews, Yiting Xu, Michael Kirberger, Jenny J. Yang

**Affiliations:** 1 Center for Diagnostics and Therapeutics, Department of Chemistry, Georgia State University, Atlanta, GA 30303, USA; candrews29@student.gsu.edu (C.A.); yxu0415@gmail.com (Y.X.); 2 Chemistry Division, Georgia Gwinnett College, Lawrenceville, GA 30043, USA; mkirberger@ggc.edu

**Keywords:** calmodulin, CaMBP, peptide, IQ motif, prediction, SVM, random forest, machine learning

## Abstract

Calmodulin (CaM) is an important intracellular protein that binds Ca^2+^ and functions as a critical second messenger involved in numerous biological activities through extensive interactions with proteins and peptides. CaM’s ability to adapt to binding targets with different structures is related to the flexible central helix separating the N- and C-terminal lobes, which allows for conformational changes between extended and collapsed forms of the protein. CaM-binding targets are most often identified using prediction algorithms that utilize sequence and structural data to predict regions of peptides and proteins that can interact with CaM. In this review, we provide an overview of different CaM-binding proteins, the motifs through which they interact with CaM, and shared properties that make them good binding partners for CaM. Additionally, we discuss the historical and current methods for predicting CaM binding, and the similarities and differences between these methods and their relative success at prediction. As new CaM-binding proteins are identified and classified, we will gain a broader understanding of the biological processes regulated through changes in Ca^2+^ concentration through interactions with CaM.

## 1. Introduction

Calmodulin (CaM) is an intracellular Ca^2+^-binding protein (CaBP) in eukaryotic systems, which functions as a second messenger that regulates myriad vital biological processes through interactions with more than 300 target proteins and peptides. The CaM protein, encoded by three different genes [1], is primarily expressed in eukaryotic organisms, and is one of most highly conserved protein sequences, with only histone proteins H4 and H3, actin B, and ubiquitin exhibiting greater evolutionary conservation. Genetic mutations to the CaM sequence can lead to various and potentially fatal pathologies, including ventricular tachycardia [2], congenital arrhythmia [3], and long QT syndrome [4]. These mutations can alter CaM’s affinity for binding Ca^2+^ and its target proteins and peptides, thus interfering with their downstream activity.

**Figure 1:**
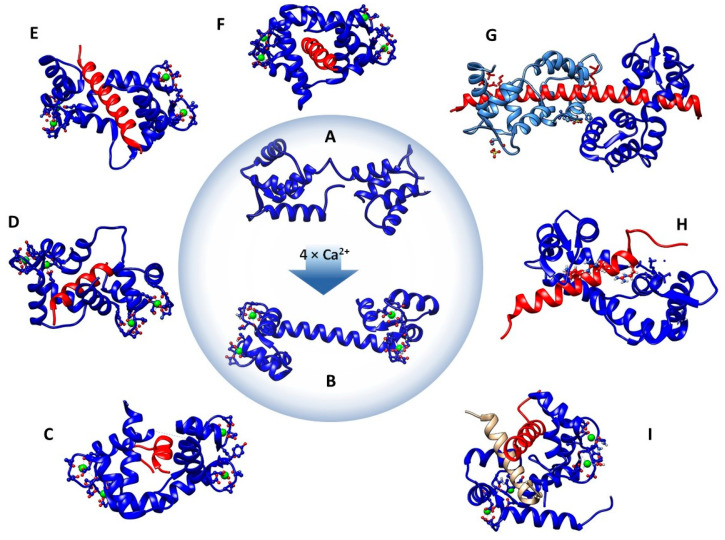
All CaM structures shown in blue, and CaMBPs are shown in red or tan. (**A**). Apo-CaM (PDB ID 1cfd) with central helix unwound/extended. (**B**). Holo-CaM (PDB ID 4bw8) with 4 Ca^2+^ ions in EF-Hand sites 1–4. (**C**)**.** Ca^2+^/CaM/MARCKS (myristoylated alanine-rich C kinase substrate) complex (PDB ID 1iwq). The N-lobe of CaM is not involved in binding [10]. (**D**). Ca^2+^/CaM/CaMKIIα complex (PDB ID 1cm1) exhibiting 1-5-10 binding mode with collapsed CaM [11]. (**E**). Ca^2+^/CaM/RyR1 complex (PDB ID 2bcx) exhibiting unusual 1-17 binding mode with collapsed CaM [12]. (**F**). Ca^2+^/CaM bound to peptide analog of CaM-binding region of chicken smooth muscle myosin light-chain kinase (PDB ID 1cdl) [13]. (**G**). Apo-CaM/Myosin V 2:1 complex (PDB ID 2ix7). Each CaM C- lobe is partially open to grip the first part of the IQ motif (IQxxxR), while the closed N-terminal lobes interact weakly with the second part of the motif (GxxxR) [14]. (**H**). Apo-CaM (blue) bound to zebrafish IQCG protein (red) (PDB ID 4lzx), exhibiting lower affinity than the Ca^2+^-bound state [15]. Sidechain interactions with CaM include residues highlighted in bold from the IQCG sequence 400-410 (**LQ**AWW**RG**TMI**R**). (**I**). Ca^2+^/CaM/Glutamate decarboxylase chains B and C (PDB ID 1nwd) [16].

Structurally, CaM is a predominantly helical protein (Figure 1A) that can be divided into N- and C-terminal domains. Each domain includes two paired Ca^2+^-binding EF-hand motifs (Figure 1B). Each motif consists of a canonical helix-loop-helix (HLH) structure. The EF-hand motif, exhibiting pentagonal-bipyramidal geometry, includes a highly-conserved sequence of 12 amino acid residues, identified by relative positions 1–12. Six of these provide oxygen atoms as the preferred ligands for coordination of Ca^2+^ ions [5] from side chains of residues in relative positions 1, 3, 5, and 12 [6], with oxygen from a carbonyl group in position 7. In addition to oxygen ligands from the amino acids, water molecules participate in forming the Ca^2+^-stabilizing coordination complex [7]. The two domains are connected by an extended helix (Figure 1B) that is observed to be partially unwound and coiled in the Ca^2+^-free state of the protein (Figure 1A) [8]. It has been experimentally verified that the extended helix has a propensity to be inherently disordered, increasing the overall flexibility of the protein, and allowing CaM to achieve different conformational states in its interactions with other peptides (Figure 1C–I) [9].

In the cell, CaM responds rapidly to increases in Ca^2+^ concentrations, which normally ranges from approximately 10–100 nM, by binding up to four Ca^2+^ ions in its paired EF-hand sites. The EF-hand pairs interact cooperatively [17], and it has been proposed that cooperativity between two coupled EF-hand binding sites of domains of CaM, causes global conformational changes in CaM that are conducive to binding target effector proteins to CaM [18]. The affinity range for each allosteric site has dissociation constant (K_d_) values between 10^−7^ and 10^−11^ M, indicating high affinity binding [19]. In general, the C-terminus motifs have a greater affinity for Ca^2+^ than for the N-terminus motifs [20,21]. Affinity equilibriums at each binding site, in addition to being sensitive to allosterically-induced conformational changes, are also responsive to whether or not CaM has formed a binding complex with another protein. These changes are binding protein specific. Thus, there is a significant, cooperativity-mediated sensitivity to Ca^2+^ that is both inherent to CaM’s native conformation and influenced considerably by intraspecific and interspecific factors.

The Ca^2+^/CaM complex alters the conformation of CaM to interact with CaM-binding domains (CaMBDs) of target proteins and peptides [22]. It is through interactions with numerous CaM-binding proteins that CaM regulates diverse physiological processes that include memory formation [23], muscle contraction [24], cellular metabolism [25], and cytoskeletal rearrangements [26]. The proteins that CaM interacts with have been found in many different cellular locations and physiological environments. Myosin light-chain kinase [24], calcineurin [27], and CaM-dependent kinases I–IV [28] are cytosolic calmodulin effector proteins involved in motility, protein dephosphorylation, and protein phosphorylation processes, respectively. Recent rapid developments in structural, genomic, and analytical methods have revealed the important roles of CaM in regulating membrane proteins, including Na_v_1.2/1.5 channels [20,21], IP_3_R channels [29], connexins [30], and GPCRs. These membrane embedded proteins participate in cellular depolarization, intracellular second-messenger signaling, and paracellular signaling, respectively.

In this review, we will first provide an overview of different CaM-binding proteins, the motifs through which they interact with CaM, and shared properties that make them good binding partners for CaM. We will then review prediction algorithms that utilize sequence and structural data to predict regions of peptides and proteins that can interact with CaM. Historical and current methods for predicting CaM binding, and the similarities and differences between these methods and their relative success at prediction, will also be discussed.

## 2. Structural Aspects and Binding Modes of CaM-Binding Proteins

The properties of CaM that facilitate its binding modes include the flexible central linker domain [31], methionine-rich linker domain, helix-helix movement, and side chain rearrangements [32]. The N-lobe of CaM participates in binding more effectively during complex formation associated with increases in local concentrations of Ca^2+^, while the C-lobe participates more effectively at lower Ca^2+^ concentrations. Thus chelation of Ca^2+^ initiates Ca^2+^-dependent conformational changes at these lobes through α-helix movement and rearrangements of side-chain contacts, which in turn affects interactions with CaM-binding proteins.

Recent work on calmodulin’s different binding complexes [33] has improved our current understanding of the mechanisms that result in the two CaM termini domains exhibiting different characteristics in binding complex modes. Despite this, a comprehensive understanding of exactly how CaM can interact with such a large number of proteins and peptides has yet to be established [34]. The use of spectral clustering system modeling, which evaluates the extent of binding (e.g., loose binding vs. compact binding) as a function of solvent exposure (hydrophobicity) and interhelical angles, led to three significant discoveries. First, shallow binding occurs more often in the Ca^2+^-free (apo) forms of calmodulin, which results in more interactions between polar and charged residues on the calmodulin-binding protein interface. Secondly, the C-terminus of the calmodulin protein has very fixed conformations for protein binding that usually lead to more compact binding modes. Interestingly, the C-terminus of CaM typically exhibits higher Ca^2+^ binding affinity than the N-terminus of calmodulin, which strongly suggests that the C-terminus holoproteins represent an intermediate mode for calmodulin. Similar to the apoprotein form of calmodulin, the third discovery demonstrated that the N-terminus of calmodulin is flexible and binds more loosely to calmodulin-binding proteins. It is likely that the low calcium and high calcium binding modes for calmodulin (apo and N-terminal, respectively) bind with lower affinity to more effectively adjust to changes in Ca^2+^ concentration. Thus, CaM may bind targets either in its apo or holo forms, and because CaM is divided into two lobes (N- and C-terminal lobes), CaM may also functional in a partially saturated state, where not all of the four EF-Hand sites are occupied.

For CaM-binding proteins (CaMBPs), binding typically involves a disorder-to-order conformational change [22], and studies on the relationships between ion channels and CaM have revealed that structural disorder provides the flexibility required for the fine-tuned modulation needed to maintain intracellular homeostasis within the extracellular milieu [35].

Targets that bind to CaM interact through regions of positive charge, hydrophilic residues, and hydrophobicity in the helices [36]. The methionine-rich grooves in the linker domain allow for interaction with CaMBPs containing amphipathic α helices that attach to holo-CaM using a pair of hydrophobic anchors [12].

During interaction with targets, CaM may also be described as exhibiting either an extended or collapsed form. In the collapsed form, connection of two anchor residues in the CaM-binding motif may reduce the distance between the two domains from 50 Å to less than 10 Å [37]. Several variations of this anchoring pattern have been identified, where the binding domain of the CaMBP include at least two hydrophobic anchor residues. Examples of sequences containing these anchoring patterns (Table 1) include 1-10, 1-12, 1-14, 1-16 [38], 1-17 [12] and 1-10-14 [39]. An extensive review of known protein structures of CaMBPs in the Protein DataBank was previously reported by Tidow and Nissen [40]. A 1–5–10 binding mode for holo-CaM in the collapsed form, determined using peptide models, was observed with the α-subfamily connexins (Cx50p_141–166_, Cx44p_132–153_ and Cx43p_136–158_) [41,42]. In its extended mode, Ca^2+^-activated CaM interacts with myosin V Ca^2+^ channels, Ca^2+^ pumps, and SK channels (Small conductance Ca^2+^-activated potassium channels), among others [41]. CaM also interacts with myosin light-chain kinase (MLCK), which binds with a 1-14 anchoring that allows for the N and C-terminals of calmodulin to wrap around the helix (Figure 1E) [43].

The IQ motif, as seen in myosin V Ca^2+^ channels, can interact with CaM when it is Ca^2+^ free [43] or only partially saturated with Ca^2+^, and in some cases, in its holo state [21] (Figure 1G). Calmodulin-dependent protein kinase II (CaMKII) also possesses a more compact 1-5-10 hydrophobic residue anchor pattern [40] (Figure 1D). This form may have evolved because the autoinhibitory domains on these proteins require more compact binding to reactivate the phosphorylation sites on the protein. All of these different patterns (Table 1) are dependent upon the extent to which calcium binds to calmodulin, meaning that the activation, inhibition, or regulation of myriad calmodulin-interacting proteins are all Ca^2+^ concentration-dependent processes. Thus, proteins with IQ motif binding patterns are likely activated in the absence of intracellular calcium (Figure 1H), whereas proteins with auto-inhibitory domains are more likely to be activated by high concentrations of calcium. Additional insight into distinctions between apo- and holo-CaM, and interactions with IQ motifs, was recently presented by O’Day et al. [34].

The activity of the skeletal muscle ryanodine receptor (RyR1) is inhibited as a result of binding of Ca^2+^ to the C-lobe of the CaM protein (Figure 1E) [12], which facilitates binding of CaM to RyR1 at its N-terminus through residues P3614-3643 [45,46,47]. Thus, inhibition is regulated at lower Ca^2+^ concentrations. CaM also interacts with cardiac RyR2. Brohus et al. reported the use of fluorescence anisotropy to confirm binding of CaM to four CaMBDs in RyR2, where increasing Ca^2+^ concentration increased binding affinity with the CaMBD [48].

CaMBDs have also been identified through experimental data for several connexins (Cx), which are members of the family of gap junction proteins expressed in the lens of the eye. High-affinity CaMBDs have been identified in Cx43, comprising residues 138–157 [49], and in Cx44, comprising residues 129–148 [50]. Both proteins include conserved hydrophobic residues in the CaMBD sequences at relative positions 1, 5, 10 and 14, corresponding to 1-10 and 1-14 CaM-binding motifs (Table 1). Similar motifs have also been reported for other proteins, including calcineurin and myosin light-chain kinase. A CaMBD was also identified in the intracellular loop of Cx50, comprising residues 141–166 [42]. Additionally, Zou et al., first reported the Ca^2+^-dependent, direct interaction of CaM with Cx45 (connexin45) in living cells, and blocking by the CaM inhibitor, N-(6-aminohexyl)-5-chloro-1-naphthalenesulfonamide hydrochloride [30]. In this scenario, NMR studies have confirmed that the N-lobe of CaM is more involved in the binding of CaM to Cx45 than the C lobe [30].

CaM-binding proteins are also involved in neurotransmission and synaptic plasticity and strength. The activity of kinase α-Ca^2+^/calmodulin kinase II, which phosphorylates presynaptic components (e.g., synapsin I), was reported to be modulated in its autophosphorylated state by interaction with Ca^2+^/CaM, resulting in either promotion or inhibition of neurotransmitter release [51]. Xia et al., reported that the activity of small-conductance calcium-activated potassium channels (SK channels) is regulated by Ca^2+^ binding to CaM, which associates with the alpha subunits in the SK channel [52]. Minakami et al., reported Ca^2+^-dependent binding of CaM to metabotropic glutamate receptor 5 (mGluR5), a G protein-coupled receptor [53]. Protein kinase C (PKC) phosphorylates the CaM-binding regions of mGluR5; so phosphorylation and CaM binding are antagonistic regulators of this signaling process. In a subsequent study, O’Connor et al. reported binding of Ca^2+^-loaded CaM to group III mGluRs, specifically the C-tail of mGluR 7 [54], which is necessary for release of G protein βγ subunits during presynaptic glutamatergic neurotransmission.

In later studies, Huang et al. reported experimental evidence of a CaMBD comprising residues 871–898 in the C terminus of Ca^2+^-sensing receptor (CaSR) [55]. CaSR, like mGluR, is a member of the family C of G protein-coupled receptors, and a putative CaMBD in CaSR was predicted by the Calmodulin Target Database [56]. Results of this study suggested a Ca^2+^-dependent, 1–14-like CaM-binding mode that involves formation of a helix in the CaMBD, which is consistent with identified CaM-binding motifs (Table 1).

## 3. Predicting CaM-Binding Proteins Using Generative Models

To understand how CaMs unique characteristics allow it to bind to a vast number of protein targets in eukaryotic systems, prediction models based on known CaMBD protein structures and established motifs were initially applied [56]. These models are computational in nature, and use structural and sequential information to determine possible CaM-binding sites on query proteins, as well as proteins available in sequential proteomics databases such as NCBI, UniProt, or the Protein Data Bank (PDB), a structural proteomics database. These bioinformatic developments have helped to understand where CaM acts as an additional catalytic subunit for proteins that were not previously identified as CaM targets.

Methods for predicting CaM-binding proteins have evolved over time, based on data obtained from prior studies, and in conjunction with the development of new algorithms and computational approaches to prediction problems. Progress in the calcium sciences will require improved computational methods to identify previously undiscovered CaM-target proteins. However, to accomplish that goal, it is important to understand which bioinformatics approaches have improved our contemporary understanding of CaM-mediated catalytic mechanisms. In particular, patterns or motifs in CaM-binding proteins, as well as circumstances that promote changes to the flexible linker region of CaM and alter its conformational state for different binding modes, are essential for understanding the computational tools that are used to predict novel CaM-binding proteins. Generally, models rely on sequential and structural data to make predictions, and the individual strengths and weaknesses of using each type of data, as well as the synergy that both data categories provide together, will be discussed at length.

### 3.1. Introduction to Machine-Learning for Classification

For classifying proteins as CaM-binding target proteins or otherwise, the use of a binary classification system is implemented. This system generally uses a 2 × 2 matrix consisting of true positives (TP), false positives (FP), true negatives (TN), and false negatives (FN). Classification problems are generally a supervised learning process, where examples of CaM-binding proteins are training examples that other proteins are trained on, using various cross-validation methods such as the leave-one-out method for SVMs [57], or the design of reliability estimation tools for neural networks. Examples of these and other approaches reviewed in this paper are summarized in Table 2.

For artificial intelligence-type algorithms, receiver operator characteristic (ROC) curves are often constructed in order to display the sensitivity and the specificity of the model in question. Sensitivity is best understood as the true positive rate (TPR), represented by Equation (1).

(1) $$\mathrm{Sensitivity}\;\left(\mathrm{TPR}\right)=\frac{\sum \left(TP\right)}{\sum \left(TP\right)+\sum \left(FN\right)}=1-FNR=\;\mathrm{Recall}$$

Conversely, specificity (i.e., selectivity) is best understood as the true negative rate (TNR, Equation (2)).

(2) $$\mathrm{Selectivity}\;\left(\mathrm{TNR}\right)=\frac{\sum \left(TN\right)}{\sum \left(TN\right)+\sum \left(FP\right)}=1-FPR$$

When plotted on a receiver operator curve, specificity is represented as (1—Specificity) on the abscissa, and sensitivity is the ordinate. Depending on the prediction system, these values are optimized at thresholds that are deemed appropriate for the problems being tested. It is also notable to mention that it is ideal for receiver operator curves to maximize the value on the ordinate at the most minimal value of the abscissa. However, this is primarily at the discretion of the designer of the model. An example of a binary classification trade-off where one might choose to maximize specificity over sensitivity would be the development of a medical diagnostic for a highly infectious and fatal disease. The cost of a false negative is considerably higher than a false positive—measured in human lives saved. However, for a cheap diagnostic test usable at home, such as a pregnancy test, it might be more efficient to maximize sensitivity at the expense of specificity. False negative results are much less likely to occur than false positive results when one chooses to approach a binary classification problem in this way. One way of representing this characteristic is by taking the integral of the curve to compute the area under the receiver operator curve (AUC). A large AUC represents a robust predictive model and is a common way to compare different predictive systems to each other, especially with respect to artificial intelligence systems.

Additionally, another AUC representation that is used for classification and prediction is known as precision-recall (AUC-PR). Recall is, for all intents and purposes, the same as sensitivity (see Equation (1)). The AUC-PR models the sensitivity of a classification system against the positive predictive value (PPV), or precision, of a prediction model.

(3) $$\mathrm{Precision}\;\left(\mathrm{PPV}\right)\;=\;\frac{\sum \left(TP\right)}{\sum \left(TP\right)+\sum \left(FP\right)}$$

Modelling sensitivity against precision versus modelling it against the selectivity of any binary classification system depends on the search objective. The AUC-ROC curve will yield values that describe how well the classifier works in general, while the AUC-PR curve will yield values that describe a classifier’s predictive capacity at a baseline probability. When there are significantly more negative examples than positive examples, it may be more useful to use the AUC-PR values, as this elucidates the relevance of true positive examples against all examples that are identified as positive (i.e., both true and false positives). Datasets of this nature are known as unbalanced datasets. One can conclude from this logic that for a balanced dataset, where there are equal amounts of positive and negative examples for binary classification, an AUC-ROC curve better represents the classification efficacy than an AUC-PR curve.

### 3.2. Prediction Using Profile Hidden Markov Models

The Calmodulin Target Database (CTD), developed by Yap and Ikura [56], is considered to be the current “gold standard” for CaM-binding protein prediction, as exemplified by their use in other CaM protein prediction databases as a standard for comparison [44,58]. This database utilizes Profile Hidden Markov Model (pHMM) algorithms for predictions, which have proven effective in the case of predicting CaM-binding sites, because they incorporate probability distributions of all possible sequences on a residue-by-residue basis. Only one sequence, however, is observed by the user—giving pHMMs their eponymous “hidden” characteristic. The CTD’s code prunes sequences that are considered improbable due to their lack of congruency to expected residue characteristics present in the CaM-binding motifs by using these pHMMs. Low probability emissions, or “misses”, are constrained by boundaries inherent to the algorithm, and these inherent boundaries are the high probability predictions, or “hits”, that are considered to be CaM-binding sites based on their similarity to the training datasets of proteins known to bind to CaM [59]. For example, if a peptide sequence matches the 1-10 motif perfectly, it will still be considered a miss if it does not contain the 3 consecutive basic residues preceding the hydrophobic anchor residue at residue 1.

pHMMs determine the optimal sequence by using a 20-residue “sequence walk” procedure when making these 1–9 integer assignments. Sequences stretching from 10–20 amino acid residues that earn scores between 7 and 9 are considered a “hit”, and thus match one of the patterns inherent to these motifs with high probability. This scoring approach uses dynamic programming [60], which compares two amino acids at a time, and assigns each residue an identifier such as “match”, “mismatch”, “insertion”, or “deletion”. Predicted matches are assigned a score of 1, and predicted mismatches are assigned a score of 0. To understand dynamic programming, it is useful to imagine a 20 × 20 mathematical matrix created from these data, where one axis represents all 20 possible amino acids and the other represents the transition associated with a position along the 21-residue sequence typical to CaM-binding proteins (since there are n-1 transitions, there would be 20 of these transitions). Certain hypothetical pathways are more likely than others in this matrix, and are determined by how similar the transition probabilities formed by this matrix are to the known CaM-binding motifs. Insertion and deletion identifiers introduce penalty parameters to account for noise in the sequence signal that originates from mismatch codon sequences at the pre-translational level. There are also other classifiers that optimize the transition probability parameters, described below.

BLAST and FASTA, two algorithms incorporated into the CTD, are responsible for identifying the motif and/or profile that matches sequences within a query protein [61]. In the context of identifying whether a prospective binding partner is a hit or a miss, the CTD incorporates many of the known motif patterns that have been experimentally verified, such as the Ca^2+^-independent IQ motif, and the Ca^2+^-dependent 1-5-10 motif (Table 1). Furthermore, by performing a multiple sequence alignment function on known protein sequences across different proteins, the motifs are given profile identities [62]. These approaches are very important for identifying and describing CaBPs and CaMBPs, as there remain numerous Ca^2+^ concentration-dependent structural conformations for CaM-modulated proteins that have yet to be resolved by NMR, circular dichroism (CD) spectroscopy, X-ray crystallography, or other structural-determination strategies. It is useful to be able to reference structural information for pruning false positives or forming new hypotheses, but sequential data are more reliable—provided that the protein has a rigid structure and a high degree of evolutionary conservation at a genomic level (i.e., fewer mutation events). These are all important considerations for interpreting the results of bioinformatics searches at a protein level.

Certain data arise from the integration of aligned motif sequences into profiles. For example, conserved residues are identified and given more probabilistic weight, and penalties for likely insertions or likely deletions stemming from mismatch codon sequences are introduced into the probability functions to account for these exceptions. However, the only way to optimize penalty parameters is to evaluate the true statistical significance of penalties, since profiles are often arbitrary, and are only capable of being optimized using manual trial-and-error methods [59]. Additionally, since conserved residues often descend from common phylogenetic ancestors, there is a capacity to inadvertently introduce bias into the prediction functions using profiles alone.

The pHMMs, such as SMART and PFAM, provide a solution to the inherent bias issue that using profiles as a prediction tool alone, do not. Each residue and sequence in a profile are treated as if the model itself did not create it, in either pHMM [59]. Both SMART and PFAM allow for penalty parameters to be determined empirically, and thus, evaluated for statistical significance without the phylogenetic bias previously described. Most importantly, these models match the profiles to putative domains that have been annotated with a wide variety of detail-specific classifiers, such as genetic variants (i.e., isoforms), active sites, phosphorylation sites, and non-catalytic binding sites (e.g., inhibitory, feedback regulators) [63]. However, there are nuances between the two pHMMs when performing these functions. PFAM is a much more conservative pHMM than SMART. SMART allows for subfamily classification and assigns putative domain boundaries to the entire domain rather than investigating annotated domain fragments unlike the PFAM system, whose domain boundaries are inherent to each seed alignment [63].

Where SMART and PFAM find common ground is through the addition of another level of statistical scrutiny when searching for matches between query proteins entered by the user, and motifs embedded in the profiles. They annotate proteins in the SWALL database according to their functional domain identities, and match motifs in the profiles to proteins in the database. Domain-specific identifiers, such as “channel” or “catalytic”, provide functional context that allow for more robust CaM-binding protein prediction. There are three verified CaM-functional mechanisms: (1) relief of autoinhibition with the CaM-binding site adjacent to, or within the autoinhibitory domain of the CaM target; (2) active-site remodeling, and; (3) CaM-induced dimerization [64]. SMART allows for the CTD to be more sensitive to functional domains that are coherent with these known CaM activation mechanisms by using terminology from gene ontology (GO) enrichment tools [65]. It should be noted that domain-sensitive pHMMs are not usable for query proteins whose physiological functions are unknown or unpublished.

The Calmodulin Target Database (CTD) derives 176 known full-alignment CaM-binding sequences using the SWALL database, which integrates search results from SWISSPROT, TREMBLE, and TrEMBLE New. Although the CTD mostly relies on 2D sequential data, it does include limited 3D structural data from known CaM-binding proteins such as the skeletal muscle myosin light-chain kinase [66], the smooth muscle myosin light-chain kinase [13], Ca^2+^/CaM-dependent protein kinase IIα [67], Ca^2+^/CaM-dependent protein kinase kinase [38], and the plasma membrane Ca^2+^ pump [68]. The CTD also utilizes biophysical parameters including hydrophobicity (Kyte–Doolittle values) [69], hydrophobic moment (Kyte–Doolittle values as a function of the Eisenberg equation) [70], and the propensity for a residue to contribute to an α helix (Chou–Fasman values) [71] to provide context-dependent predictive power for each residue.

A total of 1260 seed sequences (from the 176 full sequences) that are homologous to sequences in one of the four motif classes were identified from these proteins. Using the dynamic programming approach on this set of proteins, the CTD predicted 24 1-10 motifs, 43 1-14 motifs, 75 IQ class motifs, and 34 motifs in its miscellaneous subclass, including the 1-12, 1-16, and basic motifs. Of these 34, 16 were unclassified, but are still used in predictions in case more query proteins are found to be homologous to these in the future [56].

Various functions, such as the CTD search function that matches a query protein or query genomic sequence to a motif family, access these pHMMs to make the prediction problem less complex. These algorithms also provide a domain-based analysis of the query peptide using known nucleotide codon sequences. By checking the seed alignment information against the full alignments, the CTD incorporates multiple parameters (i.e., biophysical, structural, sequential) to make context-dependent predictions by using known information about where one might expect a motif to be on a query sequence. For example, the CaM-binding motifs are usually found immediately after the catalytic domains for CaM kinases and phosphoryl kinases, and after the channel domains for NMDA receptors, and both are close to the C-terminus of the peptide. For IP3 receptors, the CaM-binding motif is typically sequentially distant from the catalytic domain, and falls closer to the N-terminus of the peptide. Some, such as MAPK/ERK pathway proteins, identify CaM-binding sequences inside the catalytic domain. These provide reference points that increase the likelihood that a “hit” exists at those points in similar query proteins [56].

## 4. Predicting CaM-Binding Proteins Using Clustering Approaches

### 4.1. Brief Introduction to Supervised Versus Unsupervised Learning

Many classifiers, such as generative classifying HMMs that have pre-labeled training sequences, and discriminative classifiers (i.e., SVM, k-NN, RF), use supervised learning. In fact, supervised learning is inherent to all classification systems. These models use training examples of known proteins to make predictions about novel query proteins to determine whether they are CaM-binding targets. HMMs are based on generative training using a joint probability distribution (P(A∩B)). This is different than a discriminative classifier, which use a conditional probability distribution (P(A|B)). The former analyzes the probability that two different events happen simultaneously (A and B), while the latter analyzes the probability of 1 event (A) occurring as a consequence of another event (B). Additionally, HMMs tend to use empirical risk minimization, while discriminative classifiers use structural risk minimization. Empirical risk minimization chooses a decision rule, defined by a mathematical function, when classifying sets of data. Conversely, structural risk minimization has two primary goals. One is to control the empirical risk from the model’s training examples, while the second goal is to regulate the ability of the decision functions to reach a particular numerical representation of risk [72]. Justino et al. provided a more in-depth review on how these risk minimization strategies are used for each classifier system.

Another approach is based on unsupervised learning, which often consists of clustering methods that look for correlations in unlabeled data. This style of learning requires human supervision for making inferences. Since some HMMs require the comparison of sequence-based predictions to the known NMR or X-ray crystal structures for CaM-binding proteins, the HMM is sometimes considered to be a mixture of both supervised and unsupervised learning. The next section includes a discussion on a clustering method used for predicting CaM-binding protein targets. These use an a priori probability distribution (p_x_(x)).

### 4.2. Prediction Using Canonical Motif Clustering

The presence of an overlapping binding motif can improve the probability of a true positive in the data, compared with a single isolated motif. Developed by the University of Massachusetts, this prediction strategy, engineered by Mruk et al. [44], is available online as the Calmodulation and Meta-Analysis Predictor. In the associated publication regarding the search engine for calmodulation [44], the authors elaborate on the site-specific approach for identifying and predicting calmodulin-binding sites. For this study, 48 known CaM-binding proteins, and 52 sequences containing CaM-binding motifs were used as inputs to the model. The use of positive and negative weights, or bags, were applied to different binding-site windows across the protein sequence. Additionally, a motif score was assigned for each amino acid residue on the chain being analyzed. This score represented how many different canonical calmodulin-binding motifs were identified from the dataset provided. The authors input 16 different motifs, but some of the motifs were subfamilies of a parent motif class (e.g., 1-8-14 and basic 1-8-14), and were tested separately in this case, in order to avoid overgeneralizing the dataset. The authors also introduced several restraints on their algorithm to avoid false positives—for example, a window size of 10 amino acids, an average motif count of ≥2, a net electrical charge of ≥1, and an average hydrophobicity value ranging between 3 and 2.5. Results reported from the use of these biophysical parameters as filters indicated that the net electrical charge discriminator was the most useful filter for correctly identifying motifs for calmodulin binding.

Similar to the Calmodulin Target Database, there are some structural data that are also incorporated into the Calmodulation Meta-Analysis Database. The structural data, however, are not as closely tied to biophysical parameters, such as the Chou–Fasman α helix propensity values used in the Calmodulin Target Database [71]. Instead, the CaM crystal structure is matched against known crystal structures for any query protein that is also located in the PDB in order to determine whether there is a strong or weak likelihood that the query protein binds to CaM. Although there are biophysical constraints put upon the datasets used in the Calmodulation Meta-Analysis Database, these are applied at the sequence-level of prediction that is primarily used to make predictions. Most of the structural tools used in the prediction are used to prune incompatibilities between sequence and structure research findings.

**Figure 2:**
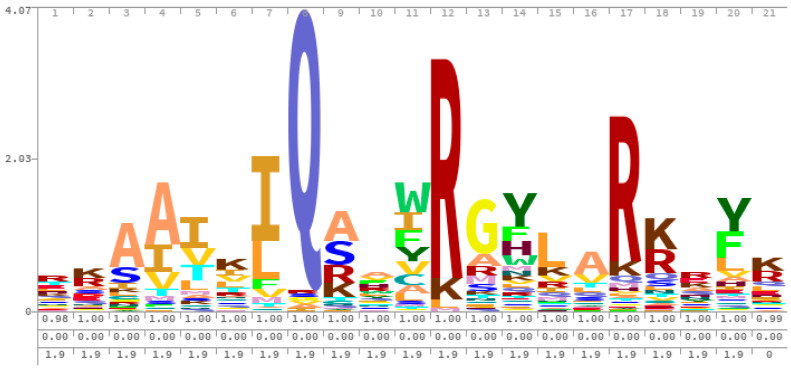
HMM logo as a visualization of profile HMM for IQ motif, from EMBL-EBI, family IQ (PF00612). The IQ motif is identified by letters at positions 7 and 8 in the logo, where increasing height of the letter represents greater distribution of the amino acid in the sequences analyzed through multiple sequence alignment.

One consideration that the Mruk approach took into account concerned the presence of multiple overlapping calmodulin-binding sites that occur frequently in known calmodulin-binding proteins. Depending on the protein in question, these sites can work synergistically to enhance calmodulin-binding. For example, the KCNQ channel protein was discovered to possess a glutamine (Q) in position 2 of an IQ motif (Figure 2) present in the protein, which is also a part of a 1-12 motif [73]. These overlapping motif data are detected by matching all of the motif patterns within a particular window to the known motifs contained within the degenerate text-pattern matching PERL script. The probability of a CaM-binding site within this window is determined by calculating a motif score for that window. Unlike Hidden Markov Models, the regular expressions script uses inference tools to find familiar patterns in the “hidden” emissions to make better predictions. However, pHMMs better represent a true protein model by cross-referencing previous patterns where indels identified in previously analyzed sequences, are pre-labeled. Thus, pHMMs are capable of considering these variables when determining the transition and emission probabilities of each residue (i.e., state), while the PERL script primarily looks for qualitative similarity, and develops a scoring system for each window to classify a region as CaM-binding or not.

There were some limitations in the use of this strategy to successfully predict CaM-binding proteins. Compared to the Calmodulin Target Database (less than 50% true positive hit rate), the Calmodulation and Meta-Analysis Predictor was more effective for predicting CaM-binding motifs in a sample protein (67% true positive hit rate), as long as the query protein entered was a smaller target (less than 100 amino acid residues). However, results were significantly worse when attempting to predict calmodulin-binding sites when the protein target was longer than 100 amino acid residues—a scenario where the Calmodulin Target Database still remains dominant in prediction power. Further, because the Calmodulation Meta-Analysis Database uses a 1D sequence-dependent strategy (i.e., degenerate text-pattern matching using the regular expressions algorithm), the potential exists for conflicting predictions when sequential or structural data are analyzed simultaneously. In fact, this is a key challenge in predicting binding domains for proteins in general. An example of this quandary arises within this database with isoform 4 of the PMCA protein. The calmodulation strategy does not predict a complex between CaM and any of the canonical motif patterns that the system recognizes. Yet, the NMR-determined binding regions of the C20 and C28 were demonstrated to show a high probability of binding to CaM, according to their Meta-Analysis.

## 5. Predicting CaM-Target Proteins Using Discriminative Models

### 5.1. CaM-Binding Protein Prediction Using Logistic Regression Classification of Disordered Proteomic Characteristics Via Neural Networks

Neural network approaches used by Radivojac and Dunker were designed to be sensitive to the intrinsic disorder inherent to many of CaM-binding proteins by classifying positive and negative examples according to features that are associated with proteins of this nature. For assigning classification labels, the neural network uses logistic regression. This method uses a sigmoid function to classify examples into positive (1) or negative (0) labels. However, this function alone is generally prone to error, identified by the term weight, and represented by the variable, *w*. A gradient descent function is introduced in order to correct for the weight contained by the sigmoid function. Another way to make the model more reliable is by reducing the cost function, which calculates the error between expected and observed values. These optimization strategies are respectively achieved using backwards propagation for the gradient descent function and forward propagation for the cost function. These are modes that help code the logistic regression neural network into programs such as Python.

Specifically, 157 unique CaM-binding proteins were assembled from the Calmodulin Target Database to test the predictive power of this approach in the context of features that are associated with proteins that are inherently disordered. Among this set of 157 proteins, there were 198 calmodulin-binding target regions, or motifs, identified [74]. The feed-forward neural network was designed to be dual layered, with one layer operating at the amino acid residue level, and a second layer working at the calmodulin-binding motif level. A total of 92 features were incorporated into prediction at the residue level, with four unique symmetric sliding windows of *w_in_* (where sequence length = 1, 7, 11, or 21). Sequence complexity, physicochemical parameters, hydrophobic moment, secondary structure, and intrinsic structural disorder, were features used as inputs in the first layer. The second layer integrates likely targets from the first layer, and then calculates probability density functions based on that data for each motif. This layer is used to form probability density functions using global indicators of calmodulin-binding related to disorder specifically, such as the flanking sequence length of the window analyzed, the percentage of predicted disorder in these flanking sequences as derived from the first layer, the globularity of the protein as a whole, and the charge-to-hydropathy ratio.

By considering emergent properties from the first layer, this devised system is well tuned to the effects of structural disorder on successful calmodulin-binding prediction. One additional self-tuning mechanism also represented and incorporated into the prediction model at the first layer is the capacity for the sequence window to expand or collapse—also known as sliding-window approach. Window frames are analyzed for their predictive power, or classification efficiency, by moving across the sequence to analyze the features when only the residues within that frame are considered. This approach is useful in response to changes in the crystal protein structure at the N- and C-termini of a protein—when it is necessary for the window to collapse to a shorter frame as a consequence of fewer amino acids available in that specific prediction frame. In this way, the positive and negative labels of CaM-target binding sites are treated computationally similar to larger windows by the model. Although the flexibility of the window being analyzed is important, each window must remain symmetrical numerically, so window sizes are often chosen to be any odd integer of amino acid residues, even at smaller window sizes.

The 92-fold feature space and the small threshold for predicted true positives regarding accurate calmodulin-binding prediction requires a machine-learning mechanism that is highly sensitive when extracting relevant parameters. Thus, 20 feed-forward neural networks with 10 hidden nodes were chosen from separate datasets as a reliability estimator to identify underrepresented or overrepresented portions of the training dataset for CaM-target binding. This model was applied to the 198 sequences in the SWISS-PROT protein sequence dataset, where sequences were then evaluated for all of the features on both levels described above. Then, each feature classifier was used as input to the feed-forward neural network, which was trained on an equal number of random data points from a positive set (e.g., the SWISS-PROT dataset) and a negative set (e.g., the calmodulin-binding protein dataset). Thereafter, the trained model’s prediction abilities were tested on each individual motif to cross-verify the efficacy of the model.

Overall, the system designed by Radivojac displayed a sensitivity of 73.2% ± 2.74 and a specificity of 88.5% ± 0.2 for prediction on an amino acid level. The average of the two predictive components indicated an accuracy percentage at the amino acid level of 80.8% and a precision of 29.1% ± 2.1. It is most notable that although the accuracy percentage between the amino acid level (i.e., first layer) and at the regional level (i.e., second layer) are comparable, the precision at the amino acid level is higher than at the regional level by 6.3% (pr = 22.8% at regional level). Many previously confirmed features that are known to be associated with a calmodulin-binding site were also confirmed by this algorithm, such as net charge and helical propensity. Some of the true findings, however, included higher *total* charge and more inherent structural disorder than non-calmodulin-binding proteins. Unstructured protein domain profiles that were found in proteins affected by calmodulin included: proteins that regulate signal shuttling between the nucleus and the cytoplasm; ribosomal proteins that also contain DNA-binding sites (likely for extra-translational activities of the ribosome); and locations that identify as repeat regions or phosphorylation sites.

### 5.2. Prediction Using Various Support Vector Machines on Arabidopsis thaliana Proteins

Profile Hidden Markov Models (pHMMs) are capable of integrating some parameters while making predictions, ranging from the sequence identity of a known calmodulin-binding motif to the biophysical parameters of the residues present in a sample sequence entered into the database. However, there are issues that limit accuracy of predictions. The Calmodulin Target Database uses the motif sequences derived from structural data as pre-labeled data that can be matched to a query protein, but it is incapable of identifying novel motif sequences. Additionally, long-distance correlative relationships between two distinct amino acids in a protein sequence are not as easily modeled as short-distance correlations, as seen when two distinct amino acids are side by side [59]. These shortcomings can increase the likelihood that a query sequence will be classified as a false positive by the CTD’s prediction system; a problem identified by the authors of the Calmodulin Target Database, resulting in a 33% false positive rate with their system [56].

To address this false positive dilemma, support vector machine (SVM) algorithms have been utilized to adjust for the complexity of a prediction system by seeking to find a linear or non-linear discriminative hyperplane. Using kernel functions, these models are able to find a space within the many dimensions of parameters that are incorporated into the model that represents “hits” (represented numerically as +1) and “misses” (represented numerically as −1) most ideally for multi-dimensional sets of data. There are several good reasons for using kernel functions to classify complex datasets. First, it allows for designers of prediction models to work with datasets that have no clear representation in a Euclidean hyperplane (i.e., linear, polynomial, exponential, etc.), which is the case with most biomolecules and their respective units (e.g., proteins to amino acids and DNA to nucleotides). Secondly, it creates decision boundaries for non-linear datasets, allowing for the application of machine learning techniques usually reserved for linear classification problems.

Kernel functions tend to solve classification problems either using polynomial or Gaussian functions applied to a multi-dimensional feature space, allowing for a strategy that classifies more complex datasets as simply as a linear classifier model can. These nuances differ between polynomial functions and Gaussian functions. For example, for polynomials, the degree (d) of the polynomial of the dot product formulation (Equation (3)) controls the flexibility of the support vector (SV)

k_d,k_^polynomial^ (x, x′) = (<x,x′> + κ)^d^ (4)

Generally, the higher-order polynomials, where d > 0, result in more flexible boundaries, but determining which one to use is a function of the datasets being analyzed. Additionally, for higher-degree polynomial functions, normalization of the function leads to better computational performance and numerical stability. Thus the use of kernel methods, using normalization to ensure that features are properly scaled, can improve AUC-ROC results with higher-degree polynomials [75].

Conversely, Gaussian functions of the dot product formulation (Equation (4)) are also useful formulations for support vector machines. Curvature of the vector space that the function occupies is important for classification.

(5) $$k_{\sigma }{}^{\mathrm{Gaussian}}\left(x,x^{\prime }\right)\;=\;\exp\;\left(-\frac{1}{\sigma }{\left|\left|x\;-\;x^{\prime }\right|\right|}^{2}\right)$$

Here, the primary variable that controls the classifier is the width parameter variable σ. When σ is small, the decision boundary for classification becomes more flexible. However, if σ becomes too small, overfitting of the datasets becomes inevitable. On the other hand, as the value of σ increases, the curvature becomes smoother, thus relaxing the boundary between positive and negative examples. If the boundary for classification becomes too relaxed (i.e., σ is too high), the Gaussian function begins to exhibit linearity, which is not ideal for classifying unbalanced sets of data. However, depending on the dataset being used, it may be useful to have a more relaxed classifier system—especially when one is optimizing a classifier for sensitivity and selectivity.

There is a tradeoff between flexibility and overfitting that is important for using kernel methods to make computational predictions. Depending on how unbalanced the dataset being analyzed actually is, it may be important to only allow certain extents of flexibility, which are commonly adjusted by computational biologists when designing kernels. In fact, with respect to designing kernels for protein–protein interactions, as is the case with CaM and its target proteins, it has proven useful to combine several kernels together to increase the performance of a classifier system [75]. CaM prediction generally uses Gaussian kernels, exhibited by the choice of classifiers used for SVM methods [58,76].

For CaM-binding protein classification, these kernels have been designed to be sensitive to different features. The 1D kernel machine function, for example, is sensitive to amino acid residues in a potential CaM-binding protein. PSI-BLAST, another kernel machine function, is sensitive to highly conserved proteins that have co-evolved in response to CaM’s ubiquitous influence on second messenger signaling. The use of different kernels to optimize the predictive capacity of a SVM has been researched extensively in context of the plant species *Arabidopsis thaliana* and its CaM-binding partners. A review by Ben-Hur et al. [75] offers an extensive elaboration regarding the mathematical specifics of many of these kernels.

Smaller cost terms found in linear SVM formulations [77] result in larger margins, and ultimately reflect a smaller penalty for misclassification by the model than larger cost terms. By having a large-margin for classification, the model is able to make predictions on examples that the model has not seen before (i.e., new query proteins). There are other points of control for the classification efficacy that depend on the type of kernel function that is being used. Generally, the width variable controls the margin for Gaussian functions, while the order of the polynomial controls the margin for polynomial functions. The margins become too flexible when smaller width Gaussian functions and higher-degree polynomial functions are used, which is similar to the cost terms in linear SVM becoming too small. These are all examples of overfitting the data. Developing the ideal predictive system using SVM will require a balance between allowing the model to handle new data, and not overfitting the data used to create the model.

Hamilton, Reddy, and Ben-Hur [64] sought to simplify the complex computational architecture of the model developed by Radivojac and Dunker by using SVM algorithms—because doing so allows for the model to apply many kernel functions to a given binary model in order to allow the model to handle a wide range of potential parameters in a multi-dimensional feature space. One way that their approach differs significantly from using a pHMM rests in the fact that the use of the sliding window approach allows for the probability of a prospective binding window to be a “hit” while not being influenced by penalty factors introduced by previous amino acid residues—particularly if these residues are not present in the window being analyzed. Thus, the SVM allows for the prediction of possible binding partners that are only subjected to the influence of a kernel function within a specific window. This strategy allows the model to recognize particular features that are influential in determining whether a potential binding site is a likely “hit”. Examples of these features are similar to the features identified by Radivojac as being critical to CaM-target binding, including helicity, charge, and aromatic content of amino acid residues.

Multiple kernels provide more sensitivity to different types of data, as previously discussed, but research indicates that this does not always lead to equal classification accuracy among all of the tested kernels. Among the kernels tested, one that is sensitive to evolutionary conservation (i.e., psi) and one that is sensitive to short motifs, like the IQ motif (i.e., gappy-pair), were calculated to have the highest AUC among all kernels (AUC = 0.88, 0.89, respectively). Additionally, when these two kernels were compared to Radivojac’s work with respect to their receiver operator characteristic, both kernels exhibited a higher maximum value of true-positive rate (e.g., y axis) at the specificity threshold of 0.026 (specificity = 0.974) than the neural network approach by Radivojac. The psi kernel yielded a value between 0.20 and 0.25 at this specificity value—representing the highest sensitivity at a considerably high specificity threshold used for their gene ontology (GO) analysis.

The SVM developed by Hamilton et al. [76] was compared to a novel SVM developed by the Minhas’ group, who extended Hamilton’s strategy by applying a SVM algorithm to a large-margin classification problem with multiple instance learning [58]. The authors developed and later applied a novel SVM algorithm of their own to improve the likelihood that a sequence is a true binding motif. The improved SVM, also known as the MI-1 SVM, considers not only the specific amino acid residues, but also the sequential coordinates of the binding motif in a designated window of N amino acid residues (i.e., a positive window, referred to as a bag), and the likelihood that the sequence will produce a hit in that specific location in the sequence by referencing coordinates in the sequence where the motif will not likely yield a match (i.e., a negative bag). This strategy is called multiple instance learning. Additionally, it presents a less complex constraint, in that at least one of the windows in a sample binding site needs to score higher than the negative windows from the same protein in order to be considered a positive window. Previously, all examples were used as the slack variables meant for training the model. In their novel MI-1 SVM, only the binding sites, or positive bags, are treated as the slack variables used for optimizing the margin between positive and negative bags. This reduces the number of variables that the algorithm needs to be trained on, and it also eliminates the need for a bias term to be introduced. The slack variables cause the boundary line to be sensitive to positive examples of CaM-binding proteins, which are merely a subset of the large number of training sets used. For example, a 6 residue IQxxxR sequence may have a greater emission probability at residues 6 through 11, than at 5 through 10. This example is within a total 21 amino acid sequence—the average number of residues in a calmodulin-binding motif. By self-referencing the test sequences, a weight system that predicts the likelihood of the motif occurring at any point in the sequence is able to improve the likelihood of a true hit, and mitigate the likelihood of a false hit. The utilization of the triplet gappy kernel, which can represent motifs within the function used for separating the feature hyperplane into positive and negative examples, allows for the model to use position-dependent information when determining whether a window contains a binding site for CaM or not. Two other kernels, the p-spectrum and the position-dependent p-spectrum, were also used to define the hyperplane between positive and negative examples. These two kernels were also combined to determine whether both position-dependent and position-independent features had a measurable impact on predictive power of the SVMs.

In addition to testing an improved version of the same algorithm used by Hamilton et al., the Minhas group also tested an improved computational strategy against the one used by Hamilton. While earlier work only used a discriminant function strategy based on maximum discriminant function score across all windows in a protein to make CaM predictions, Minhas and his group added another layer of refinement to the previous strategy, using cascaded classification. This approach helped to filter out any noise (i.e., increase the sensitivity) by defining the most probable CaM-binding sites in all positive examples within a window that are known to bind to CaM, as well as negative windows that are known not to bind to CaM. In this way, it becomes possible to filter out any non-ideal contributions to the Gaussian distribution chosen for this approach stemming from non-CaM-binding proteins—ultimately leading to more accurate CaM-binding prediction.

Although the experimenters chose 210 proteins from the Calmodulin Target Database to test the algorithm, only 153 proteins with 185 binding sites were selected, in order to fit the criteria that no two proteins have more than 40% identical sequence identity, and no two binding sites have more than 50% identical similarity in respect to CaM-binding motifs. The AUC and ROC values for the discriminative function approach were verified with a “leave-one-protein-out” (LOPO) strategy. This optimized the receiver operator curve by testing the predictive strength of the model through observation of the corresponding changes in the AUC and ROC values to a cut-off at the first 10% of false positives (AUC_0.1_) to elucidate the number of true positives produced at low false positive rates. Between each iteration of the LOPO, a 5-fold cross-validation was used to help choose the cost function, C, for the SVM. For the cascaded classification approach, a 5-fold stratified cross-validation with nested grid search was used for optimization. Specifically, different Gaussian widths and cost function values, C, were tested on the model’s examples using this approach.

As summarized in Table 3, the MI-1 SVM using the position-dependent features was more successful (AUC = 96.8, Max Std. 0.14) at increasing the likelihood of a true positive than the vanilla SVM with the position-dependent features (AUC = 95.6, Max Std. 0.16). The smaller standard deviation with the MI-1 SVM represents a smaller variability in the training samples of the MI-1 SVM, as a consequence of changes in the data, than the vanilla SVM using the previously described cross-validation methods. Improved fitting of the sample data using cascaded classification was believed to help in avoiding the overfitting of the test data, and considered the possibility that a motif might have an increased probability of being a CaM-binding site based on relative location. However, the ROC-AUC for cascaded classification, using the position-dependent feature representation, only yielded an accuracy one percentage point higher than the discriminative function prediction.

In this way, it became conceptually possible to expand the number of possible CaM-binding motifs that exist by adding a position-dependent element to where the motif has a greater likelihood of binding to CaM compared to its relative position in other parts of the same window. The MI-1 SVM exhibited the ability to classify and define new subcategories for different pre-existing classes by defining the windows within the window where motifs are more likely to exist. However, one considerable shortcoming to the Minhas’ SVM is that it only includes sequence-based information in its prediction model. Despite the novel ability of MI-1 SVM, it does not consider the whole-scale protein influence on the likelihood that a protein binds to CaM. Examples of whole-scale protein influence on prediction include 3D structure of the protein in its native conformation, as well as the general bioenergetics of the protein itself.

To this end, Abbasi and Minhas [78] innovated another CaM-binding prediction system that also uses a large-margin classification problem approach with multiple instance learning. However, their new approach, called the Calmodulin Interaction Learning System (CaMELS), is designed differently from MI-1, which uses a discriminate function system (DFS), in that it uses features extracted from the whole protein to make predictions, whereas MI-1 only looks at the most probable binding window to make predictions. Compared to MI-1, CaMELS significantly improved accuracy [79], which can help guide the identification of CaM-binding proteins and binding sites experimentally. Thus, protein–protein interaction (CaMELS) and binding site prediction (DFS) in this approach are treated as separate problems, and thus are assigned different classifiers. CaMELS is also designed to handle the imprecisions in gene ontology (GO) annotations in a manner that SVM based strategies are not designed to perform. For interaction, tertiary and quaternary structural features are used as classifiers, while secondary structural features are used for binding-site prediction using the sliding-window method.

Only small modifications were made to the binding-site window prediction system in this iteration of the algorithm—in which a stochastic subgradient algorithm is incorporated into MI-1 in order to improve the computational simplicity and, thus, the overall speed of the algorithm. Feature representations containing kernels for amino acid composition (AAC), amino acid position (PDC), physiochemically similar amino acid substitutions (Blosum), emergent physiochemical features stemming from amino acid sequential order (PD-Blosum), sequence-derived structural features (propy) and motif sequence identities (PDGT), are used at the binding-site prediction level. Similarly, kernels sensitive to amino acid composition and physiochemical properties were mostly used for the protein-level prediction analysis as well, but kernels sensitive to sequential similarity in the known CaM-binding protein training set (*n* = 157) were also utilized. The logic of implementing this kernel in prediction is rooted in the idea that structural similarity at the global proteomic level implies that two proteins will also share a similar function—lending to the problem of predicting CaM interactors.

Many types of analyses and validation methods were used to test the performance of the CaMELS method including GO enrichment, independent dataset validation analysis, in silico mutation analysis, and 10-fold stratified cross-validation. New representations of accuracy used were the AUC of the precision–recall (PR) curve, which is useful when the number of positive examples are significantly outnumbered by the number of negative examples. Additionally, a method called “rank of the first positive prediction”, or RFPP, provides bioinformaticians with a metric that ranks the top true positive predictions across all proteins. Both of these metrics provide different insights of accuracy—the former referencing hit–miss ratios, and the latter drawing conclusions about predictive accuracy by comparing the predictive power of the model with the highest rank with the most probable CaM-binding site.

Using the AUC-PR metric, there was a 43.5% increase from the predictive performance of the MI-1 SVM algorithm (58.3% vs. 14.8%) using CaMELS. With the AUC-ROC_0.1_ metric, there was a 21.2% increase between MI-1 and CaMELS performance (59.0% vs. 80.2%). The leading hypothesis as to why this system fairs much better in performance than the previous MI-1 SVM developed by Minhas is that the whole sequence of the protein provides a more robust predictive system than merely observing the binding sites. This is not surprising when one considers that CaM-binding proteins normally have multiple CaM-binding sites across the protein sequence. However, one caveat to this logic proposed by Abbasi is that due to significant sequential diversity across eukaryotes, using motif-based methods are not ideal. Even if a motif is likely to be a binding site, if it is not energetically or structurally accessible, it will not participate in a binding event with CaM. This is verified by the PR curve results where motif-based approaches (using the gappy-triplet sequence kernels) only have a 1.0% precision rate using CaMELS. So far, CaMELS appears to represent the most successful design of an artificial intelligence system with respect to CaM-binding protein prediction accuracy (Table 4). Interestingly, within the RFPP representation, the first 8 ordered binding sites yielded by the model were bound to possess the true CaM-binding site on a protein. Thus, Abbasi’s group proposed that with the CaMELS model as a guide, a maximum of only 8 wet-lab experiments must be performed to validate a prospective CaM-binding target’s binding site using their model.

### 5.3. Prediction Using Random Forest Models and k-Nearest Neighbor

#### 5.3.1. Classifying Short Linear Motifs (SLiMs) for CaM-Binding Protein Classification Using Various Classification Methods

Contrary to the argument against motifs as a prediction tool, Abbasi [78] and Li [80] attempted to use short linear motifs (SLiMs) as a means for predicting CaM-binding sites. These types of motifs differ from the CaM-binding motifs as they contribute to folding upon binding of CaM to a CaM-binding motif. Thus, their prediction systems specifically targeted these SLiMs located within structurally-disordered regions of CaM-binding proteins, specifically to avoid energetically and structurally unfavorable motif regions. Their algorithm of choice, known as the Multiple EM for Motif Elucidation (MEME), discovers novel motifs by using Expectation Maximization, or EM. This optimizes the statistical parameters, while a statistical sequence model determines the identity and width of motif sequences. SLiMs are identified both from positive and negative sets separately (SM) and clustered together (CM) in their prediction model. They are scored using both the SWS_PPM and the SWS_RE methods.

The difference in these methods is nuanced. SWS_PPM will score each prospective binding site in window increments regardless of how likely that the window in question is a binding site. SWS_RE scores the site only if it matches the regular expression script for the motif that is being searched. Three different classifications methods are primarily used from these scoring strategies for predicting whether a SLiM is a binding site or not. These classifiers include SVM, random forest, and k-nearest neighbor (k-NN). RF is used to rank the features that are extracted from the scored data, while all three are used to classify the features, in general. Clustering both positive and negative examples together proved to be the most efficient way of predicting known CaM-binding sites, especially when used with the random forest (RF) approach using the S scoring matrix (generally, the S-matrix for scoring uses the entire population of samples, while the T-matrix uses a random sampling method). On the other hand, the k-NN classifier [81] from the SM method best classified predicted CaM-binding motifs, in general, regardless of whether they were previously known or unknown. The accuracy for this method was 80.6%, despite the fact that CM had better accuracy using all classifiers overall. Additionally, the S-matrix proved to yield better accuracy than the T-matrix did across all classifiers.

#### 5.3.2. Random Forest Modelling Hot Spot Regions from Alanine-Scanning Mutagenesis for CaM-Prediction

There is a growing body of evidence suggesting that an amino acid residue’s free energy of binding plays an important role in discerning which sequences are binding sites for CaM. Binding free energy, represented as Gibbs free energy (i.e., ΔG), represents an equilibria state where an amino acid residue within a peptide contributes a significant amount of energy that is required for protein–protein binding interactions. The propensity for a residue to contribute to binding within a peptide is considerably non-uniform, and few residues among the entire peptide sequence will therefore contribute to a protein’s binding free energy. Alanine scanning mutagenesis, a technique that involves mutating sequence residues for the simplest, chiral amino acid alanine, allows for the native conformation of the wild type protein to be used as a standard for measuring that residue’s binding free energy contribution. Thus, in order to determine whether an area is considered to be a” hot spot” for binding or not, this approach uses the thermodynamic differences in binding free energy between the native amino acid residue and a alanine-mutated peptide sequence. Using this approach, another quantity that can be measured by walking an amino acid sequence is the ΔΔG value for each individual residue after mutation. Large changes in binding free energy between wild-type and alanine mutants within a given number of consecutive residues likely reflects a binding “hot spot”.

Wang and Chen’s group sought to use the change in binding free energy as a novel approach to calmodulin-binding site prediction [82]. Their system used a dataset of binding free energy data originating from 125 mutated residues in 18 protein complexes. Using their random forest (RF) model, when the change in binding free energy was greater than or equal to 2.0 kcal/mol, the native residue corresponding to this alanine mutation was considered to contribute to a prospective hot spot. Using the random forest model algorithm, 38 hot spots and 87 non-hot spots were identified from a set of protein residue sequences, and these sets were used to train their RF model.

Random forest models work by using the hot spots (positive bags) and non-hot spots (negative bags) from the training set to extract features from the mutated residue being inspected. In order to introduce ecological relevance into their prediction approach, the system also considered the amino acid residues neighboring the hot spot residue, referred to as intra-contact residues, as well as residues that interact with the hot spot residue in a front-to-back plane, or mirror-contact residues. Feature descriptors for each hot spot residue and its closest intra-contact and mirror-contact residues were extracted using their system. The 19 descriptors were categorized into five groups by the authors: residue category (e.g., hydrophilic, polar) and secondary structure; atom contacts and atom contact areas; residue contacts and physicochemical features; relative accessible surface area and relative side-chain accessible surface area; and lastly, depth index. For the three residues (query residue, intra-contact residue, and mirror-contact residue) inspected, a total of 57 features are analyzed for each alanine-mutated residue that has been assigned “hot spot” or “non-hot spot” labels.

Once the feature descriptors are extracted, they are entered into the RF model, and vetted for their accuracy to contribute to the accurate prediction of CaM-binding proteins. The impetus for using an ensemble classification algorithm such as RF comes from the capacity to use numerous decision trees to reduce the output variance of individual trees, which stabilizes classification stability and accuracy. Unpruned trees are trained on bootstrap samples, and the tree is pruned using the feature that exhibits the maximum decrease in the Gini index, which is defined as the area under the curve (AUC) between the receiver operator characteristic (ROC) curve and its diagonal. Majority voting of all possible trees is used after training, and thus, the most important features for determining a hot spot specifically can be extracted.

Utilizing these models, the prediction results determined that the top five most important features for determining a hot spot are the residue mass, residue polarizability, residue isoelectric point, relative side-chain accessible surface area, and non-residue specific relative accessible surface area, for each target residue. These features are agreeable with the characteristics that are known to be conducive to CaM–CaM ligand binding. Wang and Chen verified their model’s ability to identify both hot spots and non-hot spots on one of calmodulin’s target proteins, smooth muscle myosin light-chain kinase. The model identified 5 out of 6 known hot spots, and 4 out of 6 known non-hot spots. The model was more effective than other programs such as MINERVA and KFC at predicting hot spots, and was either more effective or equally as effective at predicting non-hot spots. Thus, the RF algorithm approach possesses the ability to reliably predict calmodulin-binding sites using an approach that does not include motif-based data.

## 6. Conclusions and Perspectives

Over the past 20 years, a handful of computational tools have been reported to identify new CaM-binding proteins. The CaMELS method, for example, tested their prediction model using LCa and SGS3, two proteins synthesized by *Nicotiana benthamiana* that have little sequential homology to the training dataset of proteins that were used to create CaMELS. Within the external validation dataset of proteins, many of the individual and CaM-complexed structures had already been previously determined, which allowed cross-verification of predicted binding sites with true binding sites. In general, residues contributing to a binding site according to the CaMELS prediction tool occurred within 5Å of the CaM structure in the CaM–CaM-binding target protein complex. Therefore, computational systems have demonstrated their ability to identify CaM-binding proteins sourced from novel species.

Overall, the computational approaches that have worked best in predicting protein–protein interactions have been the kernel-based SVM methods for protein–protein interactions. Kernel methods allow for the classification of high-dimensional, unbalanced datasets such as amino acid residue sequences. For protein–protein interactions in general, the combination of pairwise sequence-based kernels that evaluate k-mer frequency, motif content, and domain content, simultaneously demonstrated improved classification success over methods that evaluate proteins based on individual features [83]. The combination of several types of kernel methods for the prediction of CaM are exemplified with the CaMELs prediction model, where amino acid composition (AAC) and amino acid position (PDC) kernels are used synergistically in one of their kernel methods [78].

Integrating and feeding gene ontology (GO) information into protein–protein interaction classifiers is generally practical as well, because proteins that are not found within the same cellular compartments (i.e., nucleus, mitochondria, cytosol, ER, etc.) generally do not interact with one another [83], although, it is important to consider missing data in these annotations, as they may misguide the classifier, especially if the training dataset used contains a considerably smaller number of proteins. Lastly, evaluating proteins at different structural levels (i.e., secondary, tertiary, quaternary) has provided the most accurate classification, demonstrated by using both the discriminate function system (DFS), which considers secondary sequential information, and CaMELs, which looks at higher-order (e.g., tertiary and quaternary) protein structure similarities [78]. Within the context of methods evaluated in this review, CaMELS appears to represent the most successful design of an AI system with respect to CaM-binding protein prediction accuracy (Table 4).

Several additional aspects may be combined to improve the prediction ability of CaM-binding sites in proteins. First, identifying key residues that act as “hot spots” for contributing to binding interface energetics since it less likely that interactions between all residues in the target protein will have uniform energetic contributions to the protein–protein interface with CaM [84]. Further, the substitutions of Leu449Ala and Leu465Ala resulted in a 7-fold binding affinity decrease in CREB-binding protein (CBP)/p300 to the transcriptional activation domain (TAD) of ReIA—a crucial initiator of the NF-κB inflammatory response pathway [85]. Annotating the residues within these hot spot protein residues with some of the surface-area accessible ΔΔG values, or with alternate hydrophobicity values for membrane-soluble proteins (e.g., the Engelman hydrophobicity value system), may improve the shortcomings in CaM prediction for specific proteins. Second, there are often several phosphorylation sites on proteins that bind to CaM–CaM-target protein complexes, which allow for the fine-tuning of cellular responses to environmental cues. Thus, it would be helpful to annotate CaM-binding sequences that are subject to phosphorylation and are sensitive to physiological changes. Third, we have recently shown that the helical propensity of CaM-binding motifs from gap junction connexins contributes to their binding affinity to CaM. Connexin Cx45 with the highest helical propensity, exhibits the strongest CaM-binding affinity among three families of connexins including Cx43, Cx44, and Cx50 [30]. This is likely due to the fact that pre-formed structural elements, such as the various CaM-binding motifs with a strong α helical folding propensity, are energetically favorable to CaM-binding [86]. Fourth, electrostatic interactions in the proximity of CaM-binding sites of the target protein are likely tuning CaM-binding affinity. The formation of CaM complex often largely alter CaM’s ability to sense local calcium concentration change. It has been shown that the binding of connexin CaM-binding peptide motif results in differential increase in calcium binding affinity of two domains of CaM [42,50]. Fifth, it is important to expand CaM-binding databases by including membrane proteins that may provide additional factors tailored to specific cellular environment. Sixth, since most CaM-target protein interactions are multi-dimensional, extending the current prediction of short sequence motif to 3D structure information analysis is expected to provide comprehensive analysis of key determinants for CaM interaction. Molecular dynamic studies of CaM and its targeted proteins will also reveal ensemble of their conformational states that can be selected by calcium gradient changes under physiological conditions [87].

Computational methods are constantly evolving, and being replaced by newer approaches that are faster, more efficient, and more accurate. Machine Learning will become increasingly more sophisticated, allowing for more nuanced decision making from the program itself. Arguably, it is unlikely that any single approach will offer a universal solution to structural prediction, and that existing systems will merge computational methods for improvement, and/or will involve more comprehensive efforts to leverage existing data into predictors or filters to improve computational approaches. For example, common structural biomolecular features identified through statistical analyses have been used to create filters for identifying/predicting or designing Ca^2+^ binding sites in proteins [88,89,90,91,92]. Similarly, Abbasi et al. recently reported the combined use of 3D structural data from protein complexes and sequence data during training, as part of a Learning Using Privileged Information (LUPI) framework, to distinguish between low- and high-binding-affinity protein complexes and generate better predictions during testing when only sequence data are available [93].

As our prediction methods become increasingly more accurate and broaden our ability to predict structural aspects of CaM binding, our ability to understand the role of CaM in signaling pathways will become easier to untangle, which should lead to major improvements in the diagnosis and treatment of disease.

## Figures and Tables

**Table 1:** CaM-Binding Motifs.

Motif.	Sequence
**Ca^2+^ Dependent**	
1-10	[FILVW]xxxxxxxx[FILVW]
1-5-10	[FILVW]xxx[FAILVW]xxxx[FILVW]
Basic 1-5-10	[RK][RK][RK][FAILVW]xxx[FILV]xxxx[FILVW]
1-12	[FILVW]xxxxxxxxxx[FILVW]
1-14	[FILVW]xxxxxxxxxxxx[FILVW]
1-8-14	[FILVW]xxxxxx[FAILVW]xxxxx[FILVW]
Basic 1-8-14	[RK][RK][RK][FILVW]xxxxxx[FAILVW]xxxxx[FILVW]
1-16	[FILVW]xxxxxxxxxxxxxx[FILVW]
**Ca^2+^ Independent**	
IQ	[FILV]Qxxx[RK]Gxxx[RK]xx{FILVWY]
IQ-like ^a^	[FILV]Qxxx[RK]xxxxxxxx
IQ-2A	[IVL]QxxxRxxxx[VL][KR]xW
IQ-2B	[IL]QxxCxxxxKxRxW
IQ unconventional	[IVL]QxxxRxxxx[RK]xx[FILVWY]

Table reproduced from data provided by Mruk et al. [44]. Numbers indicate positions where hydrophobic residues are required. Residues in brackets can substitute for each other in that position. ^a^ Some motifs require Ca^2+^ for CaM binding x = any amino acid.

**Table 2:** CaM-Binding Prediction Models.

**Supervised Discriminative Classifier Models**	
Support Vector Machine Hamilton et al. (2011), Minhas et al. (2012), Abbasi et al. (2017)	Seeks to classify sequence data by maximizing the margin between positive and negative features in a Euclidean hyperplane space through various tunable dot product kernel functions
k-Nearest Neighbor Li et al. (2018)	Classification is based on a vote of the closest training sequence samples in a data set that is being classified, *k* = 1 for Li et al. (2018); guarantees the error rate is no worse than 2× the Bayes’ error rate
Random Forest Classification Wang et al. (2012), Li et al. (2018)	Uses various decision trees such that each tree depends on random vector values that (1) have the same probability distribution for all trees in the forest, and (2) are sampled independently to determine the mode of both positive and negative sequence data sets
Neural Network/Logistic Regression Radivojac et al. (2006)	One layer sorts various features into binary classification using a regression model at an amino acid level and models the positive examples into likely regions that are compared at the second level to sequences that are known to bind to CaM to yield predictions in a binary classification logistic regression model for likely binding proteins
**Supervised Generative Classifier Model**	
Hidden Markov Model Yap & Ikura (2000)	Uses related instances in the data to make predictions about a sequential event in question. It is a computational method that considers all possible transition states (in this case, amino acid residue represented by *N*) when forming a transition probability, but only forms an emission probability based on the most likely instance. In biological terms, the residue at the *N* − 1 position forms 20 different, hidden transition probabilities for the amino acid residue at the *N* position, but only the most likely amino acid residue is emitted, which is observed by the user
**Unsupervised Clustering Model**	
Regular Expressions PERL Script Mruk et al. (2014)	Identifies motifs that are experimentally verified to bind to CaM using a text degenerate-pattern matching script, and adds motif scores together within a 21-residue binding to represent windows that are likely to bind to CaM based off the number of binding motifs that are observed within that scoring window

**Table 3:** Comparison of Results for Vanilla SVM and MI-1 SVM.

Method	Features	AUC	AUC_0.1_	TH %	FH %
Vanilla SVM	1-Spec	95.5	53.9	**66**	2.6
	PD-1	95.6	54.5	64	2.5
	Comb.	95.9	55.1	65	2.1
	*Max. Std.*	0.16	0.59	2.2	0.15
MI-1 SVM	1-Spec	**96.0**	54.3	62	**2.1**
	PD-1	**96.8**	**58.5**	**72**	**1.3**
	Comb.	**96.9**	**59.0**	**75**	**1.2**
	*Max. Std.*	0.14	0.80	3.4	0.11
	*Gappy*	96.5	58.5	68	1.6

**Table 4:** Comparison of CaM-Binding Site Prediction Results.

Method (Author)	Features	AUC-ROC	AUC-ROC 0.1	AUC-PR
**20 Feed-forward neural networks** (Radivojac)	-	0.89	-	-
**SVM** (Hamilton)	1-spec	0.87	0.62	-
	psi	0.88	0.61	-
	gappy-pair	0.89	0.61	-
**MI-1 SVM** (Minhas)	1-spec	0.96	0.543	-
	gappy	0.965	0.585	-
	comb	0.969	0.59	-
**CaMELS** (Abbasi)	PD-Blosum	* 0.991	* 0.802	* 0.87
	Comb(AAC and PDC)	0.989	0.776	0.856
	PDGT	0.99	0.78	0.854
	PDC	0.984	0.762	0.841
	propy	0.98	0.747	0.812
	AAC	0.978	0.723	0.807
**Interaction prediction**				
**SVM** (Hamilton)	1-spec	0.71	-	-
	Gappy-pair	0.71	-	-
	psi	0.74	-	-
**CaMELS** (Abbasi)	Propy	* 0.867	* 0.651	* 0.55
	Blosum	0.784	0.326	0.114
	AAC	0.747	0.404	0.268
	SW	0.784	0.519	0.402

* Highlighted numbers indicate the best value using a particular feature representation.

## Abbreviations

AUC	Area under the receiver operator curve
CaBP	Calcium-binding protein
CaM	Calmodulin
CaMBP	Calmodulin-binding protein
CaMBD	Calmodulin-binding domain
CaMELS	Calmodulin Interaction Learning System
CaMKII	Calmodulin-dependent protein kinase II
CaSR	Calcium-sensing receptor
CBP	CREB-binding protein
CTD	Calmodulin Target Database
FFNN	Feed forward neural network
FN	False negative
FNR	False negative rate
FP	False positive
FPR	False positive rate
HLH	Helix-loop-helix
HMM	Hidden Markov model
K-NN	K-nearest neighbor algorithm
LOPO	Leave-one-protein-out strategy
MLCK	Myosin light-chain kinase
PDB	Protein DataBank
PPV	Positive predictive value
PKC	Protein kinase C
RF	Random forest algorithm
ROC	Receiver operator characteristic
SLiMs	Short linear motifs
SVM	Support vector machine
TAD	Transcriptional activation domain
TN	True negative
TNR	True negative rate
TP	True positive
TPR	True positive rate
